# Predictors of self and parental vaccination decisions in England during the 2009 H1N1 pandemic: Analysis of the Flu Watch pandemic cohort data

**DOI:** 10.1016/j.vaccine.2017.05.061

**Published:** 2017-07-05

**Authors:** Dale Weston, Ruth Blackburn, Henry W.W. Potts, Andrew C. Hayward

**Affiliations:** a Behavioural Science Team, Emergency Response Department Science & Technology, Public Health England, Porton Down, Salisbury, UK; b Institute of Health Informatics & Institute of Epidemiology and Healthcare, University College London, UK

**Keywords:** H1N1, Vaccination, Influenza, Health behaviour, Flu Watch, Pandemic, Protective behaviour, Parental vaccination, Childhood vaccination

## Abstract

During the 2009 H1N1 pandemic, UK uptake of the pandemic influenza vaccine was very low. Furthermore, attitudes governing UK vaccination uptake during a pandemic are poorly characterised. To the best of our knowledge, there is no published research explicitly considering predictors of both adult self-vaccination and decisions regarding whether or not to vaccinate one’s children among the UK population during the H1N1 pandemic. We therefore aimed to identify predictors of both self-vaccination decisions and parental vaccination decisions using data collected during the H1N1 pandemic as part of the Flu Watch cohort study.

Data were analysed separately for 798 adults and 85 children: exploratory factor analysis facilitated reduction of 16 items on attitudes to pandemic vaccine into a smaller number of factors. Single variable analyses with vaccine uptake as the outcome were used to identify variables that were predictive of vaccination in children and adults. Potential predictors were: attitudinal factors created by data reduction, age group, sex, region, deprivation, ethnicity, chronic condition, vocation, healthcare-related occupation and previous influenza vaccination.

Consistent with previous literature concerning adult self-vaccination decisions, we found that vaccine efficacy/safety and perceived risk of pandemic influenza were significant predictors of both self-vaccination decisions and parental vaccination decisions. This study provides the first systematic attempt to understand both the predictors of self and parental vaccination uptake among the UK general population during the H1N1 pandemic. Our findings indicate that concerns about vaccine safety, and vaccine effectiveness may be a barrier to increased uptake for both self and parental vaccination.

## Highlights

• To our knowledge, this is a first joint examination of general UK H1N1 self and parental vaccination.
• Data collected during the Flu Watch study (798 adults, 85 children) were analysed.
• Vaccine concerns and perceived H1N1 risk predicted self and parental vaccination.
• Addressing these issues in future could influence self and parental vaccination.

## 1 Introduction

The H1N1 (‘Swine Flu’) pandemic represented a significant worldwide public health emergency. Although initial fears concerning its potential severity were unfounded, the public health impact was nonetheless significant. Across the UK, at least 457 people died [17]. In England alone, there were 1700 critical care admissions, 7879 hospital admissions, and 580,000 general practice (GP) influenza-related consultations during the pandemic year [24]. The highest rates of infection and hospitalisation were observed among children [8].

Despite the overall positive UK response to the H1N1 pandemic [17], the pandemic influenza vaccination programme was less successful. The programme aimed to achieve 75% vaccine uptake among priority groups [17] but, in England, only attained uptake of 34.5% [33]. Strikingly, vaccine uptake among children between six months and five years old (the ages offered the pandemic vaccine, [17]) was only 23.6% in England during the pandemic [33]. From a preparedness perspective—given both that pandemic influenza represents the most significant risk of civil emergency to the UK [6], and the apparent risk posed to children by pandemic influenza—there is a clear need to understand factors that influenced vaccine uptake during the UK pandemic.

Internationally, there is a range of literature examining factors associated with vaccine uptake during the H1N1 pandemic (see [2] for a systematic review). However, the literature concerning specific factors influencing vaccine uptake during the 2009 UK pandemic is relatively sparse. Of 37 articles included by Bish and colleagues in their systematic review [2], only four specifically concerned UK vaccination: Myers and Goodwin [23], Rubin et al. [30], [31]; and Stokes and Ismail [37]. The two papers by Rubin and colleagues Rubin et al. [30], [31] used data from the same 36 Department of Health telephone surveys; a further paper using this dataset has since been published [15]. An additional four quantitative studies examine factors associated with H1N1 influenza vaccination [7], [27], [35], [39]. We are also aware of two qualitative studies [21], [22].

Of these nine quantitative papers, six concerned the behaviour of the general public [7], [23], [15], [27], [35], [39]. However, only four measured public vaccination uptake rather than intentions [15], [27], [35], [39]. Furthermore, only one of these papers explicitly considered factors associated with parental vaccination intentions [31]. Indeed, a further examination of Bish and colleagues’ systematic review reveals only four studies (including [31]) that specifically focused on parental vaccination behaviour (Setbon and Raude, 2010; Schwarzinger et al., 2010; Torun and Torun, 2010; cited in Bish et al. [2]). Although there is a broader literature concerning predictors of parental vaccination during H1N1 (e.g., [4], [5], [9], [16], [18], [26], [34]; see also Larson, Jarrett, Eckersberger, Smith, & Paterson for a broader systematic review of childhood vaccine hesitancy), we are unaware of any additional quantitative papers concerning predictors of UK parental vaccination during the H1N1 pandemic. Given the threat posed by a future influenza pandemic and the low uptake of vaccination in the prior pandemic (particularly among young children), further understanding of vaccine uptake among all ages of the UK general population during the H1N1 pandemic is critical.

Flu Watch was a prospective cohort study of households in England run between 2006 and 2011 to help improve understanding of influenza burden and the factors (demographic, social, and behavioural) associated with influenza transmission [11]. During the 2009 H1N1 outbreak (specifically during Spring 2010), a ‘pandemic cohort’ captured information on: (i) the clinical profile of the illness, and; (ii) the behavioural and attitudinal responses of members of the public towards vaccination and antiviral usage [11]. Where a household contained children (<16 years old), parents were asked to complete a questionnaire for themselves and questionnaires on behalf of any children. These data can therefore be analysed to determine predictors of both self and parental vaccination decisions (i.e., decisions to vaccinate oneself and/or one’s child). Priority groups for pandemic influenza vaccination changed during the course of the pandemic [17], [33]. At the time of surveying the Flu Watch ‘pandemic cohort’, those eligible for vaccination included social/healthcare workers, pregnant women, household contacts of immunocompromised people, seasonal influenza clinical at-risk groups, and healthy children aged 6 months to 5 years [17], [33]. In this paper, we use the Flu Watch pandemic cohort data to provide an initial examination of the potential predictors of both adult self- and parental vaccination decisions during the H1N1 pandemic. In particular, we were interested in addressing: (1) what were the significant predictors of H1N1 vaccine uptake among the 2009 Flu Watch pandemic cohort? (2) do these predictors differ depending on whether they concern self or parental vaccination decisions?

## 2 Method

### 2.1 Participants & design

Participants were a subset of the Flu Watch ‘pandemic cohort’ participants who: a) provided baseline demographic data for the Flu Watch cohort and completed a 16 item attitudinal survey concerning pandemic influenza vaccination during Spring 2010 (1953 of 3744 (52%) ‘pandemic cohort’ participants), and; b) reported being offered an influenza vaccine (specifically, all those responding “Yes” to the question “Have you been ***offered*** a flu vaccine since August 2009?”; 883 of 1953 (45%) of survey participants). There was a degree of uncertainty within the Flu Watch cohort around which vaccination was offered (e.g., 8% of individuals were unsure which vaccine they were offered), and all individuals who were offered the seasonal flu vaccine were eligible for the pandemic vaccine (see [17]). Participants in our sample therefore included a mix of those offered pandemic vaccine and/or seasonal vaccine.

Of these 883 participants, 798 (90.4%; sampled from 552 households) were adults and 85 (9.6%; sampled from 57 households) were children aged 0–15 years. Participants were not offered vaccination directly through Flu Watch (the H1N1 UK national pandemic vaccination programme was GP-based; [17]). Uptake of pandemic influenza vaccination (identified as those reporting “Yes” to the question “did you ***have*** a PANDEMIC flu vaccine in 2009 or 2010?”) was reported for 340 (43%) adults and 58 (68%) children. Survey responses could only be submitted upon completion of all questions. Previous influenza vaccination status was derived predominantly from self-report responses to the pandemic cohort study, in combination with information from medical records (see [11] for the full Flu Watch study cohort profile). Information on the date of pandemic vaccination was requested, but inconsistently reported by participants. Further participant characteristics are outlined in Table 1. All participants gave written informed consent (proxy consent for children). The protocol was approved by the Oxford Multi Centre Research Ethics Committee (06/Q1604/103).

**Table 1:** Demographic information for all participants in the Flu Watch pandemic cohort who were offered influenza vaccine.

Characteristic		Adults		Children	
		Offered vaccine	Vaccinated	Offered vaccine	Vaccinated
People		798	340	85	58
Households		552	216	57	35
	0–4 years	–	–	56	34
	5–15 years	–	–	29	24
Age group	16–44 years	84	54	–	–
	45–64 years	225	125	–	–
	65+ years	489	161	–	–
Sex	Male	376	161	39	28
	Female	422	179	46	30
Region	North	92	43	6	3
	West Midlands	29	11	4	4
	East & E. Midlands	334	152	39	27
	London	51	16	6	2
	Southeast	78	23	4	3
	Southwest	214	95	26	19
Prior vaccination	Vaccinated	311	273	58	57
	Unvaccinated	403	44	26	0
	Not known	84	23	1	1
Index of multiple deprivation (IMD) quintile	(most deprived) 1	24	9	1	1
	2	61	28	7	1
	3	208	85	17	14
	4	230	103	22	11
	(least deprived) 5	275	115	38	31
Ethnic origin	Non-white	5	3	4	2
	White	748	315	79	54
	Not known	45	22	2	2
Chronic condition		264	178	18	15
Pregnant [parent]		4	3	0	–
Vocation [of parent]	Professional	183	90	62	41
	Intermediate	85	34	14	10
	Routine	84	37	5	4
	Retired	391	147	0	0
	Student	25	19	1	1
	Not known	30	13	2	2
Healthcare-related occupation [of parent]	53	34	0	–	

Note: Responses for all children <16 years of age were provided by a parent.

### 2.2 Materials & procedure

Participants were asked to report their agreement with each of 16 statements relating to pandemic influenza or pandemic influenza vaccination (e.g., “I did not think I was at risk of pandemic flu”; “I was too busy/had too little time to get vaccinated”) using five-point Likert scales (*Strongly Disagree* - *Strongly Agree*). Separate surveys were completed online for each participant (adult aged ≥ 16 years or child aged < 16 years), with parents completing the survey on behalf of children. The parent was specifically asked about their own attitude towards their child being vaccinated rather than responding by proxy (e.g., “I did not think that my child was at risk of pandemic flu”; “I was too busy/had too little time to get my child vaccinated”). All attitudinal items are presented in the Supplementary information.

Additional available data included age group (0–4, 5–11, 11–15, 16–44, 45–64, 65+ years), pregnancy status, sex, geographical region of residence, quintile of deprivation, white/non-white ethnicity, chronic condition status, vocation (professional, intermediate, routine, retired, student, healthcare-related occupation) and previous influenza vaccination. In the case of child vaccination, vocation and occupation information reflects that of the parent completing the survey.

***Data reduction.*** Exploratory factor analysis using the principal-factor method was conducted separately for adults and children; this method seeks to identify the lowest number of factors that can account for variance common to a set of items [12]. Items relating to attitudes likely to be negatively associated with pandemic influenza vaccination (e.g., “I did not think that I was eligible for pandemic vaccine”) were reverse coded prior to data reduction for ease of interpretation. Examination of the correlation matrices and Kaiser-Meyer-Olkin tests confirmed the suitability of our data for exploratory factor analysis (with thresholds of >0.4 and >0.5, respectively, considered suitable on the basis of previous literature; [19], [14]). The change in gradient of scree plots (displaying eigenvalues for each factor in decreasing order) was used to guide factor extraction. Scree plots were favoured over other methods such as the Kaiser criterion, which apply rigid criteria, due to their greater suitability for exploratory analyses [10], [40]. Where the change in slope for the scree plot was not clear-cut (e.g., interpretable as either 3 or 4 factors) we examined the rotated (orthogonal varimax) factor matrices (which are easier to interpret) for both possible factor groups and selected the final number of factors on the basis of their compatibility with expert knowledge and the literature.

Cronbach’s alpha was computed to examine internal consistency for each identified factor. Provided acceptable levels of internal consistency were met (α ≥ 0.7); [25], the items loading on each individual factor were subsequently averaged to produce a single aggregate variable related to each factor for each participant. If the factor did not display sufficient internal consistency, then the items corresponding to that factor were analysed individually. Relative risks (RR) and associated 95% confidence intervals (CI) were approximated using Poisson regression models with robust variance estimates (as per [13], [36]) in preference to odds ratios because uptake of pandemic influenza vaccine was not rare (of those offered vaccination, 43% of adults and 68% of parents chose to vaccinate themselves/their child respectively).

## 3 Results

### 3.1 Factor analysis

Factor analysis produced three factors for adults’ attitudes towards pandemic self-vaccination decisions (Table 2). First, an eight-item factor broadly related to vaccine safety, testing and side-effects, and concerns regarding the impact of influenza on time-off work (α = 0.82). The item relating to concerns about “time off work/education because of pandemic flu” in adults was negatively correlated with all others in the same factor despite having been reverse coded prior to data reduction (see Data Reduction section above). The item was therefore returned to its original non-reversed state for incorporation into the aggregate variable for this factor. Second, a two-item factor related to vaccine safety and effectiveness (α = 0.82). The third factor fell below the threshold for acceptable internal consistency (α = 0.53) and so corresponding items were included individually.

**Table 2:** Rotated factor matrix for adult’s attitudes towards pandemic influenza and self-vaccination.

Item	F1	F2	F3	Uniqueness
I was worried that if I caught flu I might pass it on to others				0.94
Pandemic flu is very serious if you catch it				0.88
I did not think that I was at risk of pandemic flu^a^			0.44	0.75
I did not think that I was at high risk of complications of flu^a^			0.52	0.69
I was worried about having to take time off work/education because of pandemic flu^a^	−0.48			0.72
Pandemic vaccine is safe for me	0.41	0.68		0.36
Pandemic vaccine is effective in preventing me from getting flu		0.66		0.46
Natural infection provides me with stronger immunity^a^				0.90
I do not trust vaccines^a^	0.46			0.69
I did not think that I was eligible for pandemic vaccine^a^			0.44	0.79
My doctor recommended that I have pandemic vaccine				0.78
I have had flu vaccine before and it made me feel ill^a^	0.54			0.68
I was too busy/had too little time to get vaccinated^a^	0.50			0.64
I was concerned that the pandemic flu vaccine had not been tested enough^a^	0.73			0.37
I was concerned that the vaccine could make you feel as ill as flu does^a^	0.76			0.39
I was concerned about rare but serious side effects of the pandemic flu vaccination^a^	0.77			0.36

a Scale reversed.

Factor analysis yielded five factors relating to parental attitudes towards pandemic vaccination of their children (Table 3). Only the first factor (a six-item factor related to vaccine safety, side effects and effectiveness) met acceptable levels of reliability (α = 0.79). All other factors fell below the threshold (all αs < 0.57). Items corresponding to factors two-five were included individually for further analysis.

**Table 3:** Rotated factor matrix for parental attitudes towards pandemic influenza and childhood vaccination.

Item	F1	F2	F3	F4	F5	Uniqueness
I was worried that if my child caught flu they might pass it on to others		0.45				0.69
Pandemic flu is very serious if my child caught it			0.43			0.62
I did not think that my child was at risk of pandemic flu^a^		0.54				0.47
I did not think that my child was at high risk of complications of flu^a^				0.56		0.64
I was worried about my child missing education because of pandemic flu		0.45				0.59
Pandemic vaccine is safe for my child	0.79					0.24
Pandemic vaccine is effective in preventing my child from getting flu	0.40	0.63				0.40
Natural infection provides my child with stronger immunity^a^			−0.46			0.63
When it comes to my child I do not trust vaccines^a^	0.51					0.64
I did not think that my child was eligible for pandemic vaccine^a^			0.71			0.41
My doctor recommended that my child have pandemic vaccine				0.61		0.51
My child has had flu vaccine before and it made them feel ill^a^					0.64	0.45
My child was too busy/had too little time to get vaccinated^a^					0.51	0.65
When it comes to my child I was concerned that the pandemic flu vaccine had not been tested enough^a^	0.74					0.40
I was concerned that the vaccine could make my child feel as ill as flu does^a^	0.60					0.47
I was concerned about rare but serious side effects of the pandemic flu vaccination on my child^a^	0.71					0.47

a Scale reversed.

In order to assist with interpretation of the results, the individually included items corresponding to both self-vaccination and parental-vaccination are presented together in Table 4.

**Table 4:** Self-vaccination and parental vaccination items that did not reliably load onto factors and so were included individually for analysis.

Self-vaccination
I was worried that if I caught flu I might pass it on to others
Pandemic flu is very serious if you catch it
I did not think that I was at risk of pandemic flu [scale reversed]
I did not think that I was at high risk of complications of flu [scale reversed]
Natural infection provides me with stronger immunity [scale reversed]
I did not think that I was eligible for pandemic vaccine [scale reversed]
My doctor recommended that I have pandemic vaccine

Parental vaccination

I was worried that if my child caught flu they might pass it on to others
Pandemic flu is very serious if my child caught it
I did not think that my child was at risk of pandemic flu [scale reversed]
I did not think that my child was at high risk of complications of flu [scale reversed]
I was worried about my child missing education because of pandemic flu
Natural infection provides my child with stronger immunity [scale reversed]
I did not think that my child was eligible for pandemic vaccine [scale reversed]
My doctor recommended that my child have pandemic vaccine
My child has had flu vaccine before and it made them feel ill [scale reversed]
My child was too busy/had too little time to get vaccinated [scale reversed]

### 3.2 Predictors of pandemic influenza vaccination

Tables 5 (self) and 6 (parental) summarise the results of the univariate analysis. Uptake of pandemic vaccination was predicted by previous influenza vaccination for 88% of adult self-vaccination (RR 8.51, 95% CI 6.43–11.3) and 98% of parental vaccination.^2^ Uptake of pandemic vaccination was also significantly associated with age group for both parental vaccination and self-vaccination decisions. Uptake of pandemic vaccine among adults was almost 50% lower for those aged 65+ years (RR 0.54, 0.44–0.66), relative to 16–44 year olds. Among children, 5–15 year olds were more frequently vaccinated (RR 1.36, 1.04–1.79) relative to the under 5 s.

**Table 5:** Results of the univariate analysis examining the relationship between potential predictors of vaccination uptake and self-vaccination.

Predictor		RR for vaccination	95% CI	p value	Pseudo R^2^
	16–44 years	1.00	–	<0.001	0.02
Age group	45–64 years	0.89	0.73–1.08		
	65+ years	0.54	0.44–0.66		
Sex	Male	1.00	–	0.7	0.0001
	Female	0.97	0.83–1.14		
Region	North	1.05	0.81–1.35	0.07	0.01
	West Midlands	0.79	0.49–1.29		
	East & E. Midlands	1.00	0.83–1.21		
	London	0.67	0.44–1.03		
	Southeast	0.65	0.45–0.94		
	Southwest	1.00	–		
Prior vaccination	Vaccinated	8.51	6.43–11.3	<0.001	0.23
	Unvaccinated	1.00	–		
	Not known	–	–		
IMD Quintile	(most deprived) 1	0.90	0.53–1.52	0.8	0.001
	2	1.09	0.81–1.47		
	3	1.01	0.82–1.25		
	4	1.09	0.90–1.33		
	(least deprived) 5	1.00	–		
Ethnic origin	Non-white	1.69	0.95–2.99	0.1	0.001
	White	1.00	–		
	Not known	–	–		
Chronic condition^a^		2.31	1.99–2.69	<0.001	0.05
Pregnant		1.67	0.94–2.96	0.1	0.001
Vocation	Professional	1.00	–	<0.001	0.01
	Intermediate	0.86	0.64–1.15		
	Routine	0.91	0.69–1.21		
	Retired	0.79	0.65–0.96		
	Student	1.63	1.29–2.07		
	Not known	–	–		
Healthcare-related occupation		1.49	1.19–1.85	0.003	0.01
Behavioural factor 1		1.60	1.41–1.81	<0.001	0.02
Behavioural factor 2		1.92	1.73–2.13	<0.001	0.06
I was worried that if I caught flu I might pass it on to others		1.11	1.02–1.21	0.013	0.003
Pandemic flu is very serious if you catch it		1.19	1.08–1.30	<0.001	0.01
I did not think that I was at risk of pandemic flu [scale reversed]		1.34	1.23–1.46	<0.001	0.02
I did not think that I was at high risk of complications of flu [scale reversed]		1.28	1.19–1.38	<0.001	0.02
Natural infection provides me with stronger immunity [scale reversed]		1.16	1.06–1.25	0.001	0.01
I did not think that I was eligible for pandemic vaccine [scale reversed]		1.34	1.24–1.46	<0.001	0.03
My doctor recommended that I have pandemic vaccine		1.64	1.52–1.77	<0.001	0.10

a Some categories combined due to small numbers.

**Table 6:** Results of the univariate analysis examining the relationship between potential predictors of vaccination uptake and parental vaccination.

Predictor		RR for vaccination	95% CI	p value	Pseudo R^2^
Age group	0–4 years	1.00	–	0.02	0.01
	5–15 years	1.36	1.04–1.79		
Sex	Male	1.00	–	0.52	0.001
	Female	0.91	0.68–1.21		
Region^a^	North	0.68	0.30–1.58	0.60	0.01
	West Midlands	0.95	1.69–1.30		
	East & E. Midlands				
	London	0.68	0.35–1.33		
	Southeast				
	Southwest	1.00	–		
Prior vaccination	Vaccinated	–b		–b	
	Unvaccinated				
	Not known				
IMD Quintile^a^	(most deprived) 1	0.31	0.09–1.03	0.04	0.03
	2				
	3	1.01	0.77–1.32		
	4	0.61	0.39–0.96		
	(least deprived) 5	1.0	–		
Ethnic origin	Non-white	0.73	0.27–1.98	0.54	0.001
	White	1.00	–		
	Not known	–	–		
Chronic condition		1.30	0.99–1.71	0.06	0.005
Pregnant parent		–	–	–	–
Parent vocation	Professional	1.00	–	0.51	0.002
	Intermediate	1.18	0.83–1.68		
	Routine	1.23	0.76–1.98		
	Retired	–	–		
	Student	–	–		
	Not known	–	–		
Parent healthcare-related occupation	–	–	–	–	
Behavioural factor 1		1.68	1.38–2.04	<0.001	0.04
I was worried that if my child caught flu they might pass it on to others		1.06	0.91–1.23	0.46	0.001
Pandemic flu is very serious if my child caught it		1.02	0.85–1.21	0.85	0.0001
I did not think that my child was at risk of pandemic flu [scale reversed]		1.46	1.22–1.76	<0.001	0.03
I did not think that my child was at high risk of complications of flu [scale reversed]		1.13	0.98–1.32	0.10	0.01
I was worried about my child missing education because of pandemic flu		0.98	0.86–1.11	0.71	0.0003
Natural infection provides my child with stronger immunity [scale reversed]		1.23	1.09–1.40	0.001	0.02
I did not think that my child was eligible for pandemic vaccine [scale reversed]		0.88	0.76–1.01	0.06	0.004
My doctor recommended that my child have pandemic vaccine		1.07	0.94–1.21	0.32	0.002
My child has had flu vaccine before and it made them feel ill [scale reversed]		0.90	0.77–1.06	0.21	0.002
My child was too busy/had too little time to get vaccinated [scale reversed]		1.00	0.80–1.26	0.97	<0.001

a Some categories combined due to small numbers.

b Unable to estimate RR due to no variation in the outcome for unvaccinated individuals (i.e. perfect prediction).

Relative to professional vocations, self-vaccination behaviour was comparatively higher among students (RR 1.63 (1.29–2.07)) and lower among the retired (RR 0.79 (0.65–0.96)). Those working in a healthcare related occupation (RR 1.49 (1.19–1.85)) were more likely to be vaccinated than non-healthcare roles. Vaccination was also higher among those with one or more chronic conditions (RR 2.31 (1.99–2.69)) relative to those without a chronic condition. There were no relationships between parental vaccination decisions and any of these potential predictors; although having a chronic condition was suggestive of increased uptake (RR 1.30 (0.99–1.71)). Finally, for parental (but not self-) vaccination decisions, deprivation was associated with uptake of vaccination (*p* *=* 0.04); however, the direction of effect was not consistent across the quintiles of deprivation.

Having concerns about vaccine safety, testing and side-effects, and the impact of influenza on time-off work was associated with lower vaccination uptake (RR 1.60 (1.41–1.81) [some items scale reversed] – indicating a 1.6-fold increase in vaccination uptake per unit increase in the Likert scale), whereas belief in vaccine effectiveness and safety was associated with greater vaccination uptake (RR 1.92 (1.73–2.13)). All of the individual data items included for analysis were associated with uptake of pandemic vaccine among adults. Items relating to concerns about influenza (severe nature, spreading the infection to others, believing oneself to be at risk of infection or complications) were significantly associated with greater pandemic vaccine uptake (RRs 1.1–1.3; all items *p* ≤ 0.01). A belief that natural infection provides stronger immunity than vaccination was associated with lower uptake of pandemic vaccine, as was a belief that one was ineligible for pandemic vaccine (RRs 1.2–1.3, both *p* ≤ 0.001 [scale reversed]). Finally, having the pandemic vaccine recommended by one’s doctor was associated with greater uptake of pandemic vaccine (RR 1.64 (1.52–1.77)).

Concern over pandemic influenza vaccine safety, side effects and effectiveness was associated with less parental vaccination (RR 1.68 (1.38–2.04) [some items scale reversed]). Two of the individual parental belief items were significantly associated with having one’s child vaccinated against pandemic influenza. First, a belief that one’s child was at risk of influenza infection was associated with greater parental vaccination (RR 1.46 (1.22–1.76) [scale reversed]). Second, a belief that natural infection provides stronger immunity than vaccination was associated with less parental vaccination (RR 1.23 (1.09–1.40) [scale reversed]). Although not significant, there was an indication (*p* = 0.06) that a belief that one’s child was eligible for pandemic vaccine was associated with less parental vaccination. No other attitudinal items were associated with uptake of pandemic vaccine.

Finally, there were no significant relationships between either self- or parental vaccination decisions and pregnancy, sex, region or ethnicity (*p* *>* 0.5). The small number of pregnant women that were vaccinated in our sample (*n* = 3) is insufficient to detect significant effects. There was potential geographical variation (*p* = 0.07) in adult self-vaccination with people in the South East having lower uptake than those in the South West (RR 0.65 (0.45–0.94)).

## 4 Discussion

Taken together, decisions to vaccinate oneself and/or one’s child against H1N1 were broadly associated with: lower concerns about the safety and effectiveness of the pandemic influenza vaccine, greater perceived risk of influenza, and less belief that natural infection provides immunity. The concerns over vaccine safety/efficacy and perceived risk of influenza, in particular, proliferate through the research concerning self-vaccination during the H1N1 pandemic both domestically (e.g., [15], [23], [30], [37], [27], [39]), and internationally [2].

For childhood vaccination, the current observed relationships between perceived risk and child vaccination are consistent with Rubin and colleagues’ Rubin et al. [31] finding that perceived risk mediated their observed relationship between National Health Service (NHS) work and likely vaccine uptake [31]. However, this relationship between perceived risk and parental H1N1 vaccine uptake was not observed in a recent paper from the United States ([16]; but cf. [26]^3^; [34]). Furthermore, although we are aware of no published UK data that examines the relationship between concerns about vaccine safety/side effects and parental vaccine uptake, the relationship reported herein is consistent with international data. For instance, Bults and colleagues [4] reported fear of side effects as a primary reason for declining to vaccinate their child against H1N1 (see also [5], [34]).

Our findings in relation to demographic predictors of pandemic vaccine uptake demonstrated variable consistency with the UK H1N1 literature. For instance, our finding concerning the relationship between age and vaccination was consistent with findings that younger individuals would be more likely to accept the pandemic vaccine if offered it (16–24 year olds vs. 65+ year olds, [30]; 15–24 year olds vs. all other ages, [39]), but inconsistent with other research (e.g., [23] found no significant effect of age on intentions to vaccinate). Similarly, our finding that previous influenza vaccination was positively associated with vaccination against H1N1 was consistent with some existing UK literature [15], [31], but not others (Myers and Goodwin [23] found no relationship between previous seasonal vaccination and pandemic vaccination intentions).

Although our findings regarding chronic illness were consistent with existing literature indicating that individuals with a chronic illness are more likely to get vaccinated or accept the vaccine if offered (e.g., Han et al. [15]), we did not replicate any significant effects of gender (i.e., that men were more likely to accept or intend to accept the vaccine, [15], [39]) or ethnicity (although previous findings in the UK literature have been inconsistent, e.g., [7], [30]) on adult self-vaccination decisions/intentions. These inconsistent effects are not without precedence: in their systematic review of factors associated with H1N1 vaccine uptake (including several of the papers cited above), Bish and colleagues report mixed evidence for several demographic factors (i.e., age, socio-economic factors, and ethnicity) and vaccine uptake [2]. Further work is therefore needed to fully understand the relationship between demographic characteristics and uptake of pandemic influenza vaccination.

Given the consistency between the attitudinal factors associated with UK H1N1 self-/parental vaccination and the literature, there is clear scope to use the collective findings to strengthen the UK’s plans concerning the communication and roll out of future pandemic influenza vaccination. Although there is limited evidence as to the efficacy of community education campaigns when used alone, combining these campaign with other interventions (e.g., client reminders, expanded clinic hours, home visits) does have a demonstrable effect on vaccine coverage [3], [38]. Similarly, although there is little evidence regarding the effectiveness of parental vaccination campaigns, further research to develop interventions targeting parental perceptions regarding vaccination is recommended [32]. Given this, and on the basis of both our findings and the extant literature, we suggest that future development of pandemic influenza plans should consider the potential for multicomponent interventions that specifically target self- and parental perceptions of vaccination effectiveness/safety as well as perceived risk of oneself/one’s child contracting influenza to increase uptake of a pandemic influenza vaccination.

### 4.1 Limitations

Although our analysis does, to the best of our knowledge, represent the first attempt to explore predictors of both self- and parental vaccination decisions among the general population during the UK H1N1 pandemic, there are some methodological limitations to consider. First, the Flu Watch cohort as a whole was not representative of the English population. Specifically, this cohort under-represents young adults, non-white ethnic groups, individuals who are socially deprived, and those living in the North, the West Midlands, and London [11]. In addition, the pandemic cohort survey participants contained a relatively small parental vaccination sample. As a result, we were unable to conduct more detailed, multivariate analysis using this data. Caution is therefore advised when generalising the results reported herein to the UK population. We are also aware that worry about swine flu varied (although not greatly) over the course of the pandemic, with some decline by January 2010 (see Fig. 1 in [30]). As the pandemic cohort data was collected after this decline (in Spring 2010), we are unable to explore this effects of this potential attitudinal change on vaccine uptake within our dataset. We therefore recommend both: a) that all existing data concerning UK parental vaccination attitudes/decisions during the H1N1 pandemic be analysed and published, and; b) that attempts to collect data during any future influenza pandemic ensure greater coverage of parental attitudes towards vaccination and parental vaccination decisions across the duration of the outbreak.

In addition, although we did identify several significant predictors of vaccination, none of these individually accounted for more than 23% of variance in behaviour (prior adult vaccination). Despite this, the relationships observed between vaccination uptake and perceived risk, vaccine safety, and vaccine efficacy were broadly consistent not only with the existing UK H1N1 vaccination literature [15], [23], [30], [37], but also with the role of perceived risk and efficacy in several health behaviour models (e.g., Health Belief Model, [28], [29]; Theory of Planned Behaviour, [1]). We are therefore confident in the theoretical significance of our findings.

Finally, the international literature suggests several potential predictors of self- and parental vaccination that were not captured in the Flu Watch pandemic cohort survey. For instance, the source of information concerning pandemic influenza (e.g., official sources, [2]; national rather than local news, [18]) may be associated with self- and parental vaccination. Moreover, perceiving vaccination to be socially normative (i.e., recommended by individuals that are important to you) is associated with both self- and parental vaccine uptake (e.g., [2], [20]). Further research should therefore focus on assessing a wide range of predictors of both self- and parental-vaccination during any future UK pandemic outbreak in order to explain as much variance in vaccine uptake as possible.

## 5 Conclusions

To the best of our knowledge, the current paper represents the first attempt to explicitly consider predictors of both adult self-vaccination and parental vaccination among the UK general population during the H1N1 pandemic. Broadly, our results were consistent with the extant literature concerning UK vaccination decisions/intentions during the H1N1 pandemic. Our central findings suggest that concerns over the efficacy and safety of the vaccine as well as concerns regarding the perceived risk of pandemic influenza are critical determinants in both self-vaccination and parental-vaccination. These findings could be incorporated into the development of future interventions designed to improve both self and parental vaccine uptake among the UK population in the event of a future pandemic influenza outbreak.

## Conflicts of interest

None.

## Footnotes

2 It was not possible to estimate RR due to lack of variation in the parental vaccination decisions within strata of prior vaccination.

3 Although Painter and colleagues did observe a significant bivariate relationship between perceived susceptibility of one’s child to H1N1 (i.e., fear of children getting swine flu) and willingness to allow the child to be vaccinated, this relationship was rendered nonsignificant in multivariate analyses [26].

## References

[1] Ajzen I. (1991). The theory of planned behavior. Organ Behav Hum Decis Process.

[2] Bish A., Yardley L., Nicoll A., Michie S. (2011). Factors associated with uptake of vaccination against pandemic influenza: a systematic review. Vaccine.

[3] Briss PA, Rodewald LE, Hinman AR, Shefer AM, Strikas RA, Bernier RR, et al. The task force on community preventive services. Reviews of evidence regarding interventions to improve vaccination coverage in children, adolescents, and adults. Am J Preventive Med, 2000; 18(1 supp. 1): 97–140. doi: http://dx.doi.org/10.1016/S0749-3797(99)00118-X. 10.1016/s0749-3797(99)00118-x 10806982

[4] Bults M., Beaujean D.J.M.A., Richardus J.H., van Steenbergen J.E., Voeten H.A.C.M. (2011). Pandemic influenza a (h1n1) vaccination in the Netherlands: parental reasoning underlying child vaccination choices. Vaccine.

[5] Blyth C.C., Richmond P.C., Jacoby P., Thornton P., Regan A., Robins C. (2014). The impact of pandemic a(h1n1)pdm09 influenza and vaccine-associated adverse events on parental attitudes and influenza uptake in young children. Vaccine.

[6] Cabinet office. National risk register of civil emergencies 2015 edition; 2015. Retrieved on 31 October 2016 from https://www.gov.uk/government/uploads/system/uploads/attachment_data/file/419549/20150331_2015-NRR-WA_Final.pdf.

[7] Caress A.L., Duxbury P., Woodcock A., Luker K.A., Ward D., Campbell M. (2010). Exploring the needs, concerns and behaviours of people with existing respiratory conditions in relation to the H1N1 'swine influenza' pandemic: a multicentre survey and qualitative study. Health Technol Assess.

[8] de Whalley P.C.S., Pollard A.J. (2013). Pandemic influenza a (h1n1) 2009 vaccination in children: a UK perspective. J Paediatr Child Health.

[9] Dorell C, Yankey D, Kennedy A, Stokley S. Factors that influence parental vaccination decisions for adolescents, 13 to 17 years old: national immunization survey-teen, 2010. Clinical Pediatrics, 2012; 52(2): 162–170, doi: http://dx.doi.org/10.1177/0009922812468208 10.1177/0009922812468208 23221308

[10] Ford J.K., MacCallum R.C., Tait M. (1986). The application of exploratory factor analysis in applied psychology: a critical review and analysis. Personnel Psychol.

[11] Fragaszy E., Warren-Gash C., Wang L., Copas A., Dukes O., Edmunds W.J. (2016). Cohort profile: the flu watch study. Int J Epidemiol.

[12] Gaskin CJ, Happell B. On exploratory factor analysis: a review of recent evidence, an assessment of current practice, and recommendations for future use. Int J Nursing Stud 2014; 51(3): 511–521, doi: http://dx.doi.org/10.1016/j.ijnurstu.2013.10.005. 10.1016/j.ijnurstu.2013.10.005 24183474

[13] Greenland S. (2004). Model-based estimation of relative risks and other epidemiologic measures in studies of common outcomes and in case-control studies. Am J Epidemiol.

[14] Hair J.F., Black W.C., Babin B.J., Anderson R.E. (1995). Multivariate data analysis.

[15] Han Y.K., Michie S., Potts H.W.W., Rubin G.J. (2016). Predictors of influenza vaccine uptake during the 2009/10 influenza A H1N1v ('swine flu') pandemic: results from five national surveys in the United Kingdom. Prev Med.

[16] Hilyard K.M., Quinn S.C., Kin K.H., Musa D., Freimuth V.S. (2013). Determinants of parental acceptance of the h1n1 vaccine. Health Educ Behav.

[17] Hine D. The 2009 influenza pandemic. An independent review of the UK response to the 2009 influenza pandemic; 2010. Retrieved on 18 December 2015 from https://www.gov.uk/government/uploads/system/uploads/attachment_data/file/61252/the2009influenzapandemic-review.pdf.

[18] Jung M., Lin L., Viswanath K. (2013). Associations between health communication behaviors, neighborhood social capital, vaccine knowledge, and parents’ h1n1 vaccination of their children. Vaccine.

[19] Kaiser H.F. (1974). An index of factorial simplicity. Psychometrika.

[20] Larson HJ, Jarrett C, Eckersberger E, Smith DMD, Paterson P. Understanding vaccine hesitancy around vaccine and vaccination from a global perspective: a systematic review of published literature. 2007–2012. Vaccine 2014; 32(19): 2150–9. doi: http://dx.doi.org/10.1016/j.vaccine.2014.01.081. 10.1016/j.vaccine.2014.01.081 24598724

[21] Marcu A., Rubinstein H., Michie S., Yardley L. (2015). Accounting for personal and professional choices for pandemic influenza vaccination amongst English healthcare workers. Vaccine.

[22] McNeill A., Harris P.R., Briggs P. (2016). Twitter influence on UK vaccination and antiviral uptake during the 2009 H1N1 pandemic. Frontiers Public Health.

[23] Myers L.B., Goodwin R. (2011). Determinants of adults’ intention to vaccinate against pandemic swine flu. BMC Public Health.

[24] Mytton O.T., Rutter P.D., Donaldson L.J. (2012). Influenza A(H1N1)pdm09 in England, 2009 to 2011: a greater burden of severe illness in the year after the pandemic than in the pandemic year. Eurosurveillance.

[25] Nunnally J.C. (1978). Psychometric theory.

[26] Painter J.E., Gargano L.M., Sales J.M., Morfaw C., Jones L.M., Hughes J.M. (2011). Correlates of 2009 h1n1 influenza vaccine acceptability among parents and their adolescent children. Health Educ Res.

[27] Rizzo C., Fabiani M., Amlôt R., Hall I., Finnie T., Rubin G.J., Manfredi P., d’Onofrio A. (2013). Survey on the likely behavioural changes of the general public in four European countries during the 2009/2010 pandemic. Modeling the interplay between human behavior and the spread of infectious diseases.

[28] Rosenstock I.M. (1966). Why people use health services. The Milbank Memorial Fund Quarterly.

[29] Rosenstock I.M., Strecher V.J., Becker M.H. (1988). Social learning theory and the health belief model. Health Educ Behav.

[30] Rubin G.J., Potts H.W.W., Michie S. (2010). The impact of communications about swine flu (influenza A H1N1v) on public responses to the outbreak: results from 36 national telephone surveys in the UK. Health Technol Assess.

[31] Rubin GJ, Potts HWW, Michie S. Likely uptake of swine and seasonal flu vaccines among healthcare workers. A cross sectional analysis of UK telephone survey data. Vaccine 2011; 29(13): 2421–8. doi: http://dx.doi.org/10.1016/j.vaccine.2011.01.035. 10.1016/j.vaccine.2011.01.035 21277402

[32] Sadaf A., Richards J.L., Glanz J., Salmon D.A., Omer S.B. (2013). A systematic review of interventions for reducing parental vaccine refusal and vaccine hesitancy. Vaccine.

[33] Sethi M, Pebody R. Pandemic H1N1 (swine) influenza vaccine uptake amongst patient groups in primary care in England 2009/10; 2010. Retrieved on 28 October 2016 from https://www.gov.uk/government/uploads/system/uploads/attachment_data/file/215977/dh_121014.pdf.

[34] Steelfisher G.K., Blendon R.J., Bekheit M.M., Lubell K. (2010). The public’s response to the 2009 H1N1 influenza pandemic. New England J Med.

[35] Steelfisher G.K., Blendon R.J., Ward J.R.M., Rapoport R., Kahn E.B., Kohl K.S. (2012). Public response to the 2009 influenza A H1N1 pandemic: a polling study in five countries. Lancet Infect Dis.

[36] Stijnen T., Van Houwelingen H.C. (1993). Relative risk, risk difference and rate difference models for sparse stratified data: a pseudo likelihood approach. Stat Med.

[37] Stokes S., Ismail K.M. (2010). Uptake of the H1N1 vaccine by maternity staff at a university hospital in the UK. Int J Gynecol Obstetrics.

[38] Task Force on Community Preventive Services. Recommendations regarding interventions to improve vaccination coverage in children, adolescents, and adults. Am J Preventive Med 2000; 18(1, supp. 1): 92–6. https://doi.org/10.1016/S0749-3797(99)00121-X 10806981

[39] The Gallup Organisation. Flash Eurobarometer series #287 Eurobarometer on Influenza H1N1; 2010. Retrieved 18 January 2017 from http://ec.europa.eu/public_opinion/flash/fl_287_en.pdf.

[40] Tucker L.R., Koopman R.F., Linn R.L. (1969). Evaluation of factor analytic research procedures by means of simulated correlation matrices. Psychometrika.

