# Supplementary material for: Predictors of self and parental vaccination decisions in England during the 2009 H1N1 pandemic: Analysis of the Flu Watch pandemic cohort data
Source: Vaccine. 2017 Jul 5;35(31):3875–82. doi: 10.1016/j.vaccine.2017.05.061 (PMC5593150; doi:10.1016/j.vaccine.2017.05.061)
Supplement: Supplementary data 1 [file mmc1.docx]

Predictors of self and parental vaccination decisions in England during the 2009 H1N1 pandemic: Analysis of the Flu Watch pandemic cohort data

Dale Weston, Ruth Blackburn, Henry W. W. Potts, & Andrew C. Hayward

**Supplementary Information**

Supplementary Table 1

*Attitudes towards pandemic influenza and vaccine of adults who were i) offered an influenza vaccine, and ii) received the pandemic vaccine*

| Adult-self report | Adults who were offered influenza vaccine  N=798 | | | | | Adults who received pandemic vaccine  N=340 | | | | |
| --- | --- | --- | --- | --- | --- | --- | --- | --- | --- | --- |
|  | strongly disagree | disagree | neutral | agree | strongly agree | strongly disagree | disagree | neutral | agree | strongly agree |
| a)     Pandemic vaccine is safe for me | 1% | 3% | 32% | 49% | 15% | 1% | 0% | 14% | 57% | 29% |
| b)     Pandemic vaccine is effective in preventing me from getting flu | 1% | 5% | 42% | 41% | 10% | 1% | 2% | 28% | 51% | 18% |
| c)     Natural infection provides me with stronger immunity | 5% | 18% | 45% | 28% | 5% | 7% | 21% | 42% | 25% | 4% |
| d)     I do not trust vaccines | 27% | 44% | 21% | 5% | 2% | 36% | 47% | 13% | 2% | 2% |
| e)     I was worried that if I caught flu I might pass it on to others | 4% | 14% | 28% | 44% | 11% | 5% | 10% | 25% | 45% | 14% |
| f)      Pandemic flu is very serious if you catch it | 2% | 12% | 30% | 44% | 12% | 2% | 10% | 24% | 47% | 17% |
| g)     I did not think that I was at risk of pandemic flu | 9% | 35% | 31% | 23% | 2% | 15% | 46% | 23% | 14% | 3% |
| h)     I did not think that I was eligible for pandemic vaccine | 22% | 34% | 22% | 19% | 3% | 33% | 40% | 15% | 9% | 4% |
| i)      My doctor recommended that I have pandemic vaccine | 13% | 25% | 23% | 27% | 12% | 8% | 9% | 11% | 47% | 25% |
| j)      I did not think that I was at high risk of complications of flu | 10% | 25% | 29% | 32% | 4% | 19% | 32% | 21% | 23% | 5% |
| k)     I have had flu vaccine before and it made me feel ill | 31% | 43% | 17% | 6% | 3% | 37% | 41% | 16% | 4% | 2% |
| l)      I was worried about having to take time off work / education because of pandemic flu | 24% | 34% | 32% | 7% | 3% | 24% | 30% | 32% | 9% | 4% |
| m)    I was too busy / had too little time to get vaccinated | 37% | 42% | 18% | 2% | 1% | 47% | 36% | 14% | 1% | 1% |
| n)     I was concerned that the pandemic flu vaccine had not been tested enough | 16% | 34% | 31% | 14% | 5% | 24% | 38% | 26% | 11% | 1% |
| o)     I was concerned that the vaccine could make you feel as ill as flu does | 17% | 36% | 31% | 13% | 3% | 26% | 37% | 25% | 10% | 1% |
| p)     I was concerned about rare but serious side effects of the pandemic flu vaccination | 15% | 35% | 33% | 14% | 3% | 21% | 38% | 26% | 14% | 1% |

Supplementary Table 2

*Attitudes of parents towards pandemic influenza and vaccine for children who were i) offered an influenza vaccine, and ii) received the pandemic vaccine*

| Parental Report | Children who were offered influenza vaccine  N=85 | | | | | Children who received pandemic vaccine  N=58 | | | | |
| --- | --- | --- | --- | --- | --- | --- | --- | --- | --- | --- |
|  | strongly disagree | disagree | neutral | agree | strongly agree | strongly disagree | disagree | neutral | agree | strongly agree |
| a)     Pandemic vaccine is safe for my child | 7% | 6% | 27% | 53% | 7% | 3% | 0% | 21% | 66% | 10% |
| b)     Pandemic vaccine is effective in preventing flu in my child | 4% | 5% | 29% | 58% | 5% | 5% | 0% | 22% | 67% | 5% |
| c)     Natural infection provides my child with stronger immunity | 4% | 16% | 31% | 41% | 8% | 5% | 22% | 31% | 36% | 5% |
| d)    When it comes to my child, I do not trust vaccines | 21% | 54% | 16% | 8% | 0% | 26% | 53% | 21% | 0% | 0% |
| e)     I was worried that if my child caught flu that they might pass it on to others | 4% | 19% | 22% | 47% | 8% | 3% | 17% | 22% | 47% | 10% |
| f)      Pandemic flu is very serious if my child catches it | 2% | 5% | 15% | 66% | 12% | 3% | 3% | 16% | 64% | 14% |
| g)     I did not think that my child was at risk of pandemic flu | 16% | 59% | 16% | 7% | 1% | 22% | 60% | 17% | 0% | 0% |
| h)     I did not think that my child was eligible for pandemic vaccine | 31% | 55% | 11% | 2% | 1% | 27% | 55% | 12% | 3% | 2% |
| i)      My doctor recommended that my child have pandemic vaccine | 9% | 12% | 12% | 42% | 25% | 12% | 5% | 12% | 41% | 29% |
| j)      I did not think that my child was at high risk of complications of flu | 12% | 36% | 27% | 24% | 1% | 14% | 40% | 26% | 21% | 0% |
| k)     My child has had flu vaccine before and it made them feel ill | 26% | 53% | 19% | 1% | 1% | 22% | 55% | 19% | 2% | 2% |
| l)      I was worried about my child would need to take time off education because they got pandemic flu | 26% | 33% | 21% | 14% | 6% | 27% | 31% | 24% | 10% | 7% |
| mi) My child was too busy to get vaccinated | 49% | 44% | 6% | 1% | 0% | 49% | 41% | 10% | 0% | 0% |
| mii)    I was too busy / had too little time to get my child vaccinated | 49% | 44% | 6% | 1% | 0% | 50% | 41% | 9% | 0% | 0% |
| n)     When it comes to my child, I was concerned that the pandemic flu vaccine had not been tested enough | 7% | 24% | 27% | 28% | 14% | 9% | 31% | 29% | 26% | 5% |
| o)     I was concerned that the vaccine could make my child feel as ill as flu would | 5% | 32% | 21% | 39% | 4% | 7% | 40% | 21% | 33% | 0% |
| p)     I was concerned about rare but serious side effects of pandemic flu vaccination in my child | 4% | 21% | 34% | 33% | 8% | 5% | 28% | 38% | 22% | 7% |
